# Nod2 Mediates Susceptibility to *Yersinia pseudotuberculosis* in Mice

**DOI:** 10.1371/journal.pone.0002769

**Published:** 2008-07-23

**Authors:** Ulrich Meinzer, Sophie Esmiol-Welterlin, Frederick Barreau, Dominique Berrebi, Monique Dussaillant, Stephane Bonacorsi, Fabrice Chareyre, Michiko Niwa-Kawakita, Corinne Alberti, Ghislaine Sterkers, Claude Villard, Thecla Lesuffleur, Michel Peuchmaur, Michael Karin, Lars Eckmann, Marco Giovannini, Vincent Ollendorff, Hans Wolf-Watz, Jean-Pierre Hugot

**Affiliations:** 1 INSERM, U843, Université Paris 7, Hôpital Robert Debré, Paris, France; 2 Assistance Publique Hôpitaux de Paris, service de gastroentérologie pédiatrique, Hôpital Robert Debré, Paris, France; 3 Department of Molecular Biology; Umeå University, Umeå, Sweden; 4 ISM2/Biosciences, UMR CNRS 6263, Université Paul Cézanne, Marseille, France; 5 INRA, UMR 866, Université de Montpellier II Différenciation cellulaire et croissance, Montpellier, France; 6 Université Paris 7, EA3102, Assistance Publique Hôpitaux de Paris, service d'anatomie pathologique, Hôpital Robert Debré, Paris, France; 7 Université Paris 7, EA3105, Assistance Publique Hôpitaux de Paris, service de microbiologie, Hôpital Robert Debré, Paris, France; 8 INSERM U674, Université Paris 7 - Denis Diderot, Institut Universitaire d'hématologie, Paris, France; 9 INSERM CIC-EC, Université Paris 7, AP-HP, département d'épidémiologie clinique, Hôpital Robert Debré, Paris, France; 10 Assistance Publique Hôpitaux de Paris, Université Paris 7, service d'immunologie, Hôpital Robert Debré, Paris, France; 11 Plateau Protéomique Timone, UMR911 CRO2 UFR Pharmacie, Marseille, France; 12 Laboratory of Gene Expression and Signal Transduction, Department of Pharmacology, School of Medicine, University of California San Diego, La Jolla, California, United States of America; 13 Department of Medicine, School of Medicine, University of California San Diego, La Jolla, California, United States of America; University of Birmingham, United Kingdom

## Abstract

Nucleotide oligomerisation domain 2 (NOD2) is a component of the innate immunity known to be involved in the homeostasis of Peyer patches (PPs) in mice. However, little is known about its role during gut infection *in vivo*. *Yersinia pseudotuberculosis* is an enteropathogen causing gastroenteritis, adenolymphitis and septicaemia which is able to invade its host through PPs. We investigated the role of Nod2 during *Y. pseudotuberculosis* infection. Death was delayed in Nod2 deleted and Crohn's disease associated Nod2 mutated mice orogastrically inoculated with Y. pseudotuberculosis. In PPs, the local immune response was characterized by a higher KC level and a more intense infiltration by neutrophils and macrophages. The apoptotic and bacterial cell counts were decreased. Finally, Nod2 deleted mice had a lower systemic bacterial dissemination and less damage of the haematopoeitic organs. This resistance phenotype was lost in case of intraperitoneal infection. We concluded that Nod2 contributes to the susceptibility to *Y. pseudotuberculosis* in mice.

## Introduction

Caspase Recruitment Domain 15 (*CARD15*), which encodes Nucleotide oligomerisation domain 2 (NOD2), is a member of the phylogenetically conserved NACHT-leucine rich repeats (NLR) gene family which is implicated in the innate immune response [1]. Nod2 is an intracellular protein expressed in myelomonocytic cells and activated epithelial cells [2]–[4]. *In vitro*, muramyl-dipeptide (MDP) (a moiety of the bacterial cell wall peptidoglycane (PGN) of almost all bacteria) has been identified as the minimal bacterial motif recognized by NOD2 [5], [6]. MDP activates NOD2, leading to its interaction with the serine-threonine kinase RIP-like interacting caspase-like apoptosis regulatory protein kinase (RICK) and finally to the activation of the nuclear factor-κB (NF-κB) pathway [5]–[7]. NOD2 mutations have been associated with Crohn's Disease (CD), a Human condition characterised by a chronic or relapsing inflammation on the digestive tract (Hugot et al. 2001; Ogura et al. 2001).

Only limited data are available about the role of Nod2 during host pathogen interactions. *In vitro*, Nod2 has been found to be involved in the bacterial clearance of *Salmonella* and *Streptococcus* species [8]. *In vivo*, Nod2 deleted mice have been reported to be more susceptible to oral infection with *Listeria monocytogenes* [9] and this finding was paralleled to decreased secretion of some antibacterial cryptidins by Paneth cells [9].

The genus of *Yersinia* includes three species that are pathogenic for humans and rodents. *Y. pestis* is the causative agent of plaque. *Y. pseudotuberculosis* and *Y. enterocolitica* are enteropathogens that cause most often self limited enteritis and mesenteric adenolymphitis in Human. In some cases, especially in patients with hemochromatosis, however, enteric *Yersinia* disseminates systemically with a case fatality of 70% [10]. In mice, oral inoculation with enteropathogenic *Yersinia* results in translocation of bacteria from the intestines to spleen and liver and subsequently in the death of the animals [11].

Enteropathogenic *Yersinia* strains display a tropism to lymphoid tissue [12]. The bacteria bind to and invade M cells within the follicle-associated epithelium overlying the lymphoid follicles of the Peyer's patches (PPs) [13], [14]. Following their entry into PPs, the bacteria induce the host immune response, which is characterized by an inflammation with infiltration of neutrophils and macrophages [15].

We have previously shown that Nod2 is involved in the homeostasis of PPs [16]. Nod2^−/−^ mice are characterised by a higher number of PPs and LFs after birth. In addition, PPs are larger and contain an increased proportion of M cells and CD4^+^ T-cells and higher levels of TNFα, IFNγ, IL12 and IL4. In contrast, little differences are found in the PP-free ileum and the spleen of Nod2^−/−^ mice. Finally, PP modifications are associated with increased paracellular permeability and yeast/bacterial translocation. The aim of this study is to further explore the role of Nod2 in the function of PPs using a model of oral infection by *Y. pseudotuberculosis* in a Nod2 invalidated mouse model.

## Results

In order to determine if Nod2 plays a role in the immune defence against *Y. pseudotuberculosis* we first established oral survival curves and determined the oral lethal dose 50 (LD50) in Nod2^−/−^ mice. Five groups of five Nod2^−/−^ mice in the C57BL/6j genetic background as well as the corresponding Nod2^+/+^ control mice were orogastrically inoculated with 10-fold serial dilutions of the *Y. pseudotuberculosis* strain YPIII (pIB102) [17] (supplementary Figure S1a). The Lethal Dose 50 (LD50) was only slightly higher for Nod2 deficient mice than for wild type mice (1.4×10^7^ CFU versus 5.4×10^6^ CFU). Similar results were observed with knock-out and wild type mice in the FVB/N genetic background with LD50 values respectively of 2.7×10^6^ CFU and 5.3×10^5^ CFU (supplementary Figure S1b).

**Figure 1:**
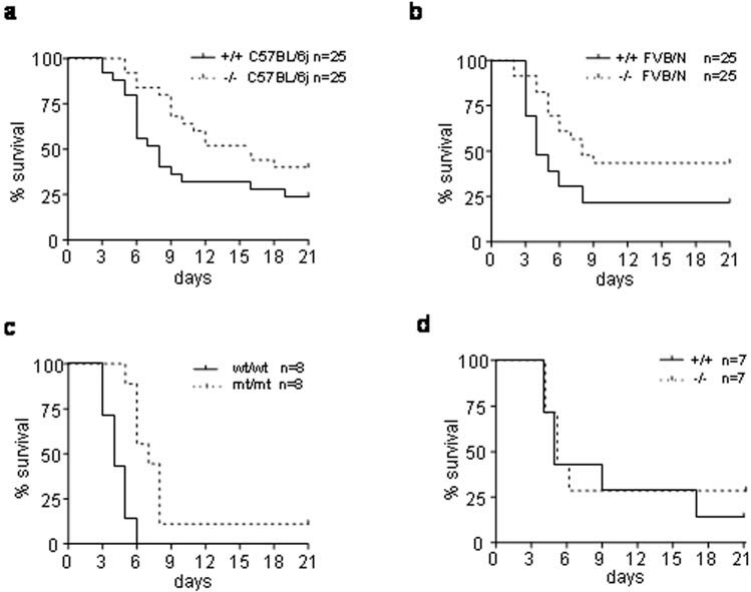
Nod2 mediates susceptibility to *Y. pseudotuberculosis* infection in mice. *Nod2^+/+^* and *Nod2^−/−^* mice in the C57BL/6j and FVB/N backgrounds were orogastrically inoculated with 10 fold dilutions ranging from 6,5×10^5^ to 6,5×10^9^ CFU of *Y. pseudotuberculosis* pIB102 wild-type strain. Survival curves of mice, all different infection dose groups used for LD50 analysis confounded, are shown for the (a) C57BL/6j background and (b) for the FVB/N background. Nod2^−/−^ mice died significantly later than Nod2^+/+^ control mice (p<0.01; Log-Rank test). (c) Nod2 mutated mice were infected with 1×10^9^ CFU of YPIII(pIB102) and died significantly later than Nod2 wild type littermate controls (p<0.001; Log-Rank test). (d) No survival difference was seen after intraperitoneal infection of Nod2^−/−^ and Nod^+/+^ mice with 5×10^4^ CFU of YPIII(pIB102) (P = 0.61; Log-Rank test).

These limited differences in LD50 values did not argue for a strong biological effect of Nod2 pathway in the host immune response. However, it appeared that for all the tested doses, the invalidated mice died later than the control mice in both the FVB/N and C57BL/6j genetic backgrounds (supplementary Figure S1). When comparing the Kaplan-Meyer curves using the Log-Rank test, we found that this difference reached significance for some inoculated doses (supplementary Figure S1) and for the pooled data (p<0.01; Figure 1a,b).

Interestingly, it is to note that the slope of the survival curves was similar in both Nod2^+/+^ and Nod2^−/−^ mice suggesting that, even if delayed, the mechanisms of death (usually due to a generalised infection) should be the same in both mouse strains. In addition, the genetic susceptibility of FVBN mice is higher than the C57BL/6 mice, confirming the importance of the genetic background in *Y. pseudotuberculosis* infection.

In order to further confirm the role of Nod2 in the susceptibility to *Yersinia* infection, we used an additional mouse model carrying the mutation homologous to the CD associated frame-shift mutation 1007fs (Nod2^mt/mt^) [18]. Here too, after oral infection with 1×10^9^ CFU of the YPIII(pIB102) strain, Nod2^mt/mt^ mice had a survival advantage when compared to littermate wild type controls (P<0.001; Figure 1c).

**Figure 2:**
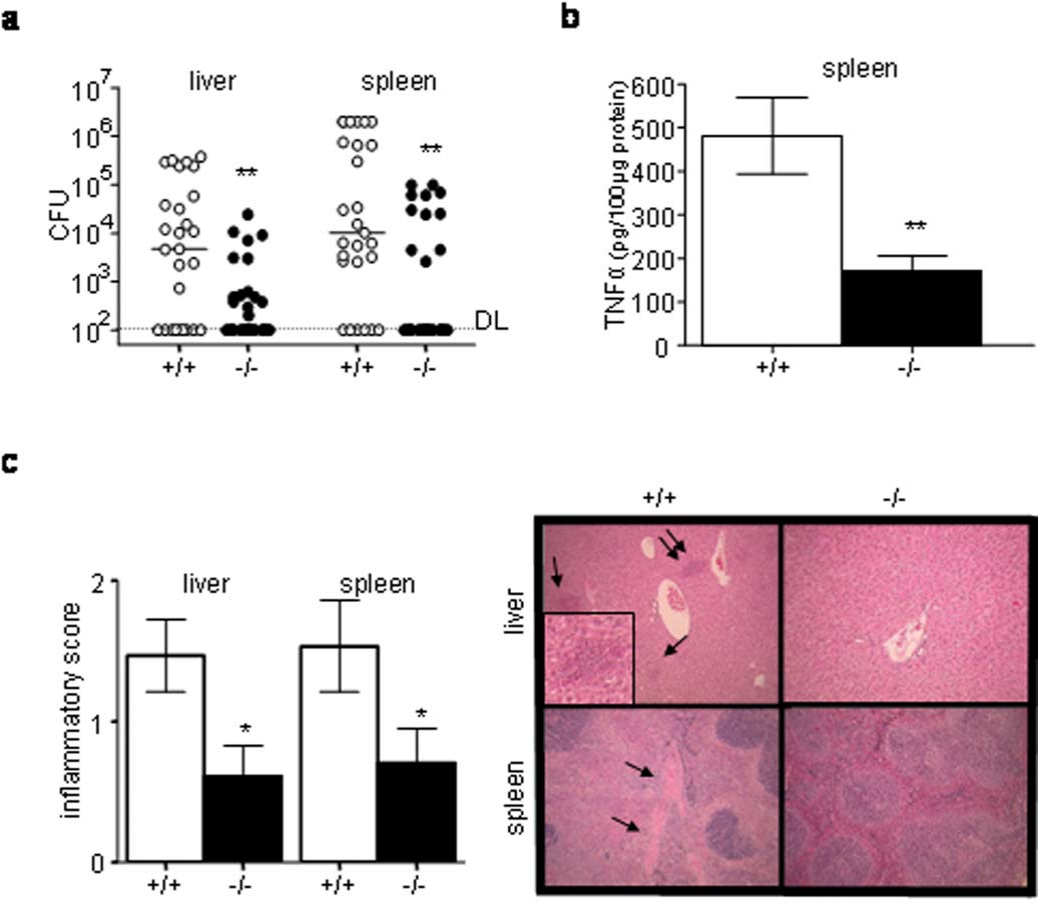
Lower bacterial counts and histological damage in organs of *Nod2^−/−^* mice (a–c) *Nod2^−/−^* and *Nod2^+/+^* mice in the C57BL/6j background were orogastrically inoculated with 1×10^7^ CFU of YPIII(pIB102) and their livers and spleens were analyzed at day 5. (a) Bacterial counts were lower in *Nod2^−/−^* (n = 29) than in *Nod2^+/+^* (n = 27) mice (Mann Whitney test, bars show medians). Detection limit was 10^2^ CFU (b) TNFα levels in spleen (ELISA) were lower in *Nod2^−/−^* (n = 6) than in *Nod^+/+^* (n = 5) mice (Student t-test) (c) Scores of histological damages were lower in *Nod2^−/−^* (n = 18) than in *Nod2^+/+^* (n = 15) mice (Student t-test). Photos show representative lesions in HE stained tissues. Arrows indicate lesions; double arrows indicate the lesion shown in higher magnification. Error bars indicate mean+/−SEM. *P<0.05, **P<0.01.

Further exploring the phenotype, we focused our work on C57BL/6j mice orally inoculated with 1×10^7^ CFU of wild type *Y. pseudotuberculosis* and sacrificed at the beginning of the fifth day following infection, when all mice inoculated with this dose are still alive. *Nod2* deficient mice had lower bacterial counts, lower TNFα levels and less intense inflammatory tissue damages in spleen and liver than wild type controls (P<0.01; Figure 2a–c). As a result, we concluded that the better survival observed in *Nod2^−/−^* mice was associated with a less intense systemic bacterial dissemination and tissue damages in haematopoietic organs.

Because Nod2 is involved in the gut immune response, we tested if the infection route influences the animal survival by infecting Nod2^−/−^ mice in the C57BL/6j background with 5×10^4^ CFU of the YPIII(pIB102) strain via the intra-peritoneal route. In contrast to oral infection, no differences were observed between Nod2^−/−^ and control mice (Figure 2d). This finding was reproduced using serial dilutions of the inoculum (data not shown). We thus concluded that Nod2 mediates the susceptibility to infection with *Y. pseudotuberculosis* via the oral but not the via the intra-peritoneal route.

We thus hypothesized that the intestinal immune response might be the key factor for the decreased systemic dissemination of *Y. pseudotuberculosis*. In agreement with previous reports [15], histological analysis of intestines showed that intestinal lesions were mainly situated in areas containing lymphoid follicles and PPs (data not shown). Consequently, we further focused on PP areas.

**Figure 3:**
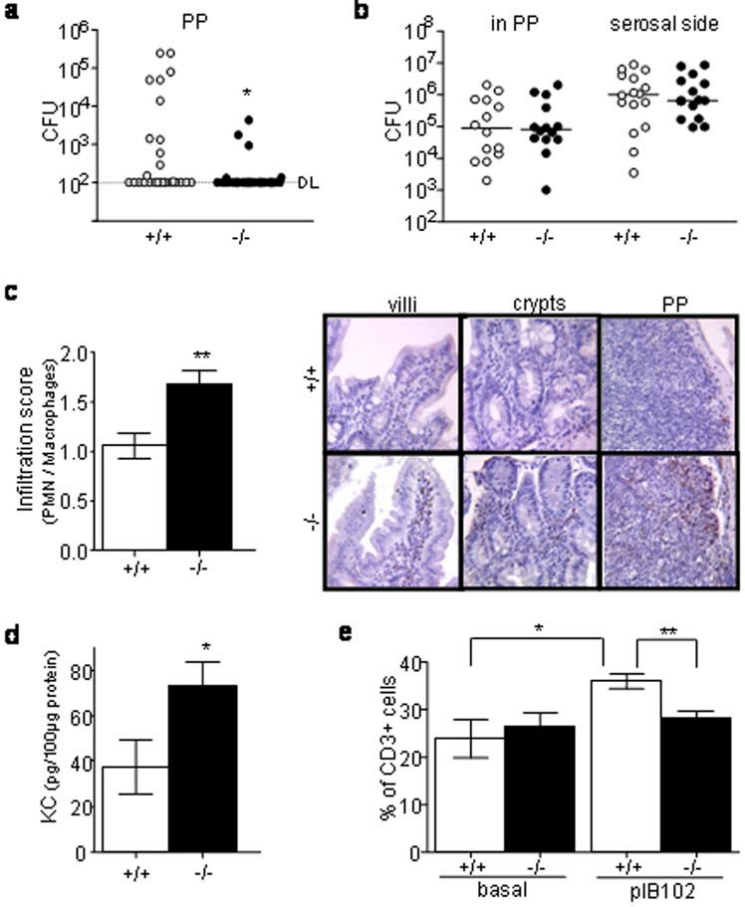
Nod2 modulates the local intestinal inflammatory response during *Y. pseudotuberculosis* infection. (a, c–d) *Nod2^−/−^* and *Nod^+/+^*mice were orogastrically inoculated with 1×10^7^ CFU of YPIII(pIB102) bacteria and sacrificed 5 days after inoculation. Their PP were removed and analyzed. (a) Bacterial counts were significantly lower in *Nod2^−/−^* (n = 29) than in *Nod^+/+^* (n = 27) mice (Mann-Whitney-test) for a detection limit (DL) of 10^2^ cfu. (b) PPs from non infected mice were placed in a Ussing chamber and 1×10^8^ CFU/ml pIB102 were placed in the mucosal compartment of the chamber. No differences between *Nod2^−/−^* (n = 7) and *Nod2^+/+^* (n = 7) mice for the bacterial translocation trough PP (mucosal to serosal flux, P = 0.85) as well as for the bacterial colonisation of the tissue (count of the bacteria remaining in the tissue after the experiment; P = 0.94) were seen at 120 min (Mann-Whitney test). Detection limit was 10^2^ CFU. (c) Infiltration of macrophages and neutrophils was scored in PPs and their surrounding epithelium. The infiltration score was higher in *Nod2^−/−^* (n = 9) than in *Nod2^+/+^* (n = 11) mice (Student t-test). Photos show representative MPO staining of intestinal villi, crypts and PPs (d) KC concentrations were recorded by ELISA. KC level was higher in *Nod2^−/−^* (n = 6) than in *Nod2^+/+^* (n = 7) mice (Student t-test). (e) Flow cytometry analyses revealed an increase of the relative proportion of CD3^+^ T-cells after infection in *Nod2^+/+^* mice (n = 6) but not in *Nod2^−/−^* mice (n = 6) (Student t-test). Error bars indicate mean+/−SEM. *P<0.05, **P<0.01, ***P<0.001.

Five days after intragastric inoculation with *Y. pseudotuberculosis*, *Nod2^−/−^* mice were characterized by lower bacterial counts in PPs (P<0.05, Figure 3a) and the number of bacteria found in PPs was correlated with the number of bacteria found in spleen and liver (P<0.01). Furthermore, the decreased bacterial colonisation of PPs was observed as early as day two after infection when little bacteria have translocated into liver and spleens (p<0.05, supplementary Figure S2). Taken together these results suggest that PP areas form a more efficient barrier in infected *Nod2^−/−^* mice.

Ussing-chambers were used to further study the *ex vivo* translocation rate through PPs. No differences between *Nod2^−/−^* and *Nod2^+/+^* mice were found, neither in terms of bacterial flux through PPs, nor in terms of bacterial colonisation of the tissue after addition of 1×10^7^ CFU or 1×10^8^ CFU in the mucosal part of the chamber (Figure 3b and data not shown). These findings suggested that bacterial translocation through PPs was determined by an active local immune response rather than by a passive passage through the gut mucosa.

Consequently, we studied the cell composition of the PPs and the bordering intestinal tissue. Under basal conditions, *Nod2^−/−^* and *Nod2^+/+^* mice showed no differences in the proportions of CD3^+^ T-cells, B220^+^ B-cells and the number of macrophages and neutrophils in the intestine (data not shown). In contrast, five days after oral infection with *Y. pseudotuberculosis*, the *Nod2^−/−^* mice were characterized by an increased inflammatory score in PPs and the surrounding epithelium (P<0.01, Figure 3c). Indeed, *Nod2^−/−^* mice had often a marqued mucosal inflammation with punctuated mucosal erosions while *Nod2^+/+^* mice had most often a mild mucosal infiltrate without ulceration. This phenotype was associated with an enhanced secretion of KC, an IL-8-homologue implicated in neutrophil recruitment (P<0.05, Figure 3d). *Nod2^−/−^* mice were also characterized by no change in the proportion of CD3^+^ T-cells in comparison to the basal (non infected) level, while *Nod2^+/+^* mice showed an increased level of CD3^+^ T-cells after *Yersinia* infection (Figure 3e). No differences were found with respect to B220^+^ B-cells in both *Nod2^+/+^* and *Nod2^−/−^* mice (data not shown).

**Figure 4:**
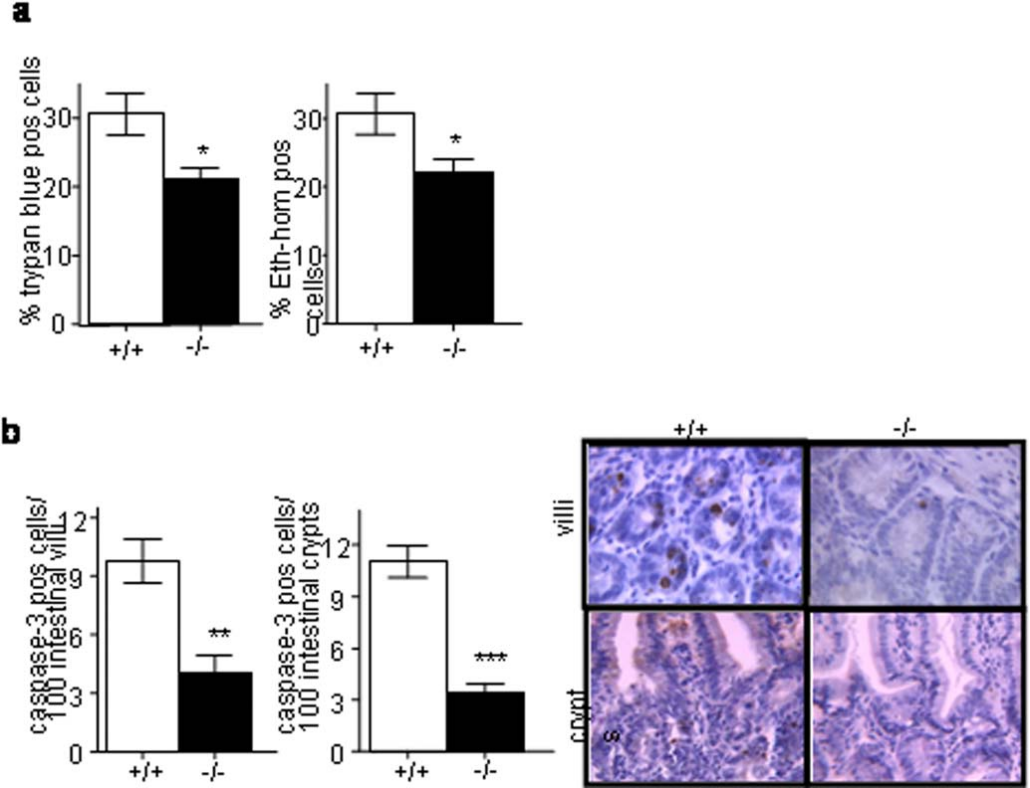
Less apoptosis of epithelial cells surrouding PP areas in *Nod2^−/−^* mice *Nod2^−/−^* and *Nod^+/+^*mice were orogastrically inoculated with 1×10^7^ CFU of YPIII(pIB102) bacteria and sacrificed 5 days after inoculation. Their intestines were removed and analyzed.(a) Cell death measured by the proportion of Trypan blue or Ethidium homodimer-1 positive cells was lower in the PPs of *Nod2^−/−^* mice (Student t-test). (b) Apoptosis measured by the number of caspase-3 stained epithelial cells in the 100 intestinal villi and crypts surrounding PPs was also lower in *Nod2^−/−^* mice (Student t-test). Photos show representative caspase-3 staining of intestinal villi and crypts. Error bars indicate mean+/−SEM. *P<0.05, **P<0.01, ***P<0.001.

Yersinia species are known to induce cell death [19]. We thus explored this parameter in the Nod2^−/−^ mice. Under basal conditions, the number of dead cells in PPs and the surrounding epithelial cells was low and there was no difference between Nod2^−/−^ and Nod2^+/+^ mice (data not shown). In agreement with the observation of a lower bacterial colonization of the gut mucosa in *Nod2^−/−^* mice, we found fewer trypan blue and ethidium homodimer-1 positive dead cells in PPs from these mice compared to control mice (P<0.05; Figure 4a). In addition, *Nod2^−/−^* mice showed significantly less cleaved caspase-3 positive epithelial cells in the intestinal villi (P<0.01; Fig. 4b) and crypts (P<0.001; Fig. 4b) surrounding PPs, suggesting that cell death induced by Yersinia infection was at least in part related with apoptosis.

Finally, we explored the *in vitro* function of macrophages and dendritic cells derived from bone marrow cells. We failed to identify abnormalities in knock out mice for TNFα or IL1β secretion, bacterial internalisation and cell survival after *Y. pseudotuberculosis* infection (supplementary Figure S3).

Altogether, these data showed that the intestinal response of PPs after oral infection with *Y. pseudotuberculosis* is impaired in *Nod2^−/−^* mice.

## Discussion

Nod2 has been identified as an intracellular receptor of the innate immune system. It is involved in the recognition of pathogen associated molecular patterns present in the bacterial cell wall but little is known about its role during the host response towards pathogenic bacteria. We have recently reported that in contact with a non pathogenic bacterial flora Nod2^−/−^ mice have an increased number of PPs which are characterized by a large proportion of CD4^+^ T-cells and increased concentrations of pro-inflammatory cytokines [16]. Here, we used *Y. pseudotuberculosis* as a model to show that Nod2 also plays a role in the immune response of PPs toward enteropathogenic bacteria.

We show that Nod2 is involved in the *in vivo* response towards *Y. pseudotuberculosis*. This phenotype is not dramatic, as indicated by the limited differences observed in the LD50 between knock out and wild-type mice. However, this result is meaningful as indicated by its reproducibility in several mouse models and in several genetic backgrounds. Interestingly, the same phenotype is observed in a knock out model and in a model of mice carrying a mutation homologous to the main Human CD associated mutation (1007 fs). This mutation is usually considered as a loss of function mutation in Human while it has been associated with an over stimulation of the IL-1β pathway in mouse [18]. It is out of the scope of this paper to fully resolve the question but it is to note that our results suggest that it is possible to conciliate the two models at least in terms of the immune response to *Y. pseudotuberculosis*.

For *Y. pseudotuberculosis* and *Listeria monocytogenes* [9], the phenotype of Nod2 deficient mice is observed only when mice are infected via the oral route. These observations suggest that Nod2 has a special role in the intestine as also indicated by the association between NOD2 mutations and CD. Indeed, the intestinal phenotype of Nod2^−/−^ mice is characterized by an increased KC secretion and a stronger infiltration by macrophages and neutrophils and finally by a lower bacterial count. In parallel with the lower bacterial cell count, we found less apoptotic damages in the mucosa and a limited bacterial dissemination associated with a better survival.

If Nod2 is a susceptibility gene for *Y. pseudotuberculosis*, it also mediates protection against *Listeria monocytogenes* [9]. In order to know if the observed resistance toward Yersinia infection could be seen with another enteropathogenic strain, we analysed the survival curves after intragastric inoculation by 5×10^8^ CFU of *Salmonella typhimurium*. In opposition to *Y. pseudotuberculosis*, *Nod2^−/−^* mice were found to be more sensitive to Salmonella infection (P<0.05; Supplemenatry Figure S4). Furthermore, *in vitro*, Nod2 has been shown to participate in the bacterial clearance of *Salmonella* [2] species and *Streptococcus* [8]. Thus, the negative role of Nod2 observed after *Yersinia* infection appears to be the exception rather than the rule.

Our data suggest that *Y. pseudotuberculosis* may interact with Nod2 signalling pathways. The ability of *Yersinia pseudotuberculosis* to multiply in PPs and to spread to deeper tissues is dependent on a plasmid that is common to all *Yersinia* species [20]. This virulence plasmid (pYV) encodes a type III secretion apparatus and several secreted proteins called Yersinia outer proteins (Yops). Given the intracellular localization of Nod2, the effector Yops seem to be good candidates when searching for bacterial components able to interact with Nod2 signalling. The Yop effectors have been shown to interfere with several signal transduction pathways within eukaryotic cells [21], paralyzing phagocytosis, cell migration, NF-κB activation and other functions that are required for innate and adaptive immune responses [22]. Further studies are now necessary to identify how *Y. pseudotuberculosis* is able to interact with Nod2 signalling.

Because NOD2 is a susceptibility gene for CD, the impact of our observations for CD patients is questionable. Several lines of evidence indicate that PPs may be important areas for the occurrence of CD lesions. A spatial relationship between CD and PPs is supported by the observation that CD lesions mainly occur in areas rich in lymphoid follicles including the distal ileum and the colon. In addition, Fujimura et al. have shown that the inaugural CD lesions are centered by lymphoid follicles [23]. A temporal relationship between CD and PPs has also been proposed considering that ileal CD is rare in young and old people when PPs are less developed [24]–[26]. Furthermore, we have previously shown that Nod2 is involved in the homeostasis of PPs [16]. We show now that Nod2 is also implicated in the PP immune response towards *Y. pseudotuberculosis* and provide hereby further indirect data in favour of a link between Nod2 and the function of PPs. Additional studies are now needed to further study the role of lymphoid tissues for the pathogenesis of CD.

It is also interesting to note that several data support a link between *Yersinia* species and CD. First, the clinical presentation of CD and Yersiniosis are both characterized by ileitis or ileocolitis with granulomas and in some cases by reactive arthritis. Second, many case reports and a case control study have shown that CD may occur after a Yersiniosis [27]. Third, mononuclear cells from mesenteric lymph nodes of CD patients are more reactive to *Y. enterocolitica* when compared to other bacteria such as *E.coli*, *Salmonella agona*, *Candida albicans* or *Chlamydia trachomatis* [28]. Fourth, *Y. enterocolitica* and *Y. pseudotuberculosis* have been found in CD lesions [29]–[31]. Altogether, these data suggest further exploring the relationship between the exposure to *Yersinia* and the occurrence of CD.

## Materials and Methods

### Mice

*Nod2^−/−^* mice [16] and Nod2^mt/mt^ mice [18] have been described previously. *Nod2^−/−^* mice were backcrossed at least 5 times with C57BL/6 or FVBN mice. Nod2^mt/mt^ mice were in a mixed C57BL/6×129 background. All mice as well as the corresponding control mice were born, generated and housed at the animal facility of Robert Debré Hospital, Paris, France. 10–14 week old mice were used throughout the study. All mice were housed in pathogen free conditions with free access to food and water. Housing and experiments adhered to current institutional animal healthcare guidelines and national legislation. All experiments were approved by the local ethics committee.

### Infection of mice

Bacterial strains used in this study were YPIII(pIB102) [17]. Bacteria from fresh selective agar plates were grown in LB over night at 26°C and resuspended in PBS. For the LD50 analysis, 25 mice were inoculated intragastrically with 10 fold dilutions ranging from 6,5×10^5^ to 6,5×10^9^ CFU using a gastric tube. Death was recorded for 21days following inoculation and LD50 was calculated using the Reed-Munch method [32]. 5×10^4^ CFU were used for intraperitoneal infection and survival was recorded during 21 days. For survival experiments of Nod2^mt/mt^ mice, animals were intragastrically inoculated with 1×10^9^ CFU. *Salmonella enterica serovar typhimurium* (strain 22676) was a clinical isolate provided by S.B. Salmonella were gown over night in LB medium at 37°C and mice were incoluated with 100 µl of 1% bicarbonate and and 30 min later intragastrically infected with 5×108 CFU.

For bacterial counts in organs and for ELISA assays, oral infections were performed with 1×10^7^ CFU. At day 5, spleen, liver and 3 PPs were removed from each mouse, washed thoroughly and homogenised in PBS containing a complete protease inhibitor cocktail (Roche). Serial dilutions were performed and plated on selective LB-Agar. For ELISA assays, proteins were quantified by protein assay (Biorad). ELISAs were performed in duplicates with commercial kits (BD Bioscience) according to manufacturer's instructions.

### Histology and Immunohistochemistry

Spleens, livers and small intestines were fixed in 4% phosphate buffered formalin and stained with haematoxylin and eosin. Intestinal sections were scored for inflammatory infiltration of neutrophils and macrophages and inflammatory lesions as followed: 0: No lesion; 1: Mild mucosal inflammatory infiltrate without ulceration; 2: Marked mucosal inflammation with punctuate mucosal erosions, 3: Prominent inflammation with ulceration extending through the muscularis mucosae. In liver and spleen, inflammation was scored as followed: 0: no lesion; 1: rare and small microabcesses. 2: frequent and/or large non confluent lesions 3: confluent lesions with destruction of architecture.

Single-label immunohistochemistry was performed using ABC kit (Vector laboratories). Briefly, 4 µm deparaffinised sections were subjected to a heat-induced antigen recovery in sodium citrate buffer solution pH6. Endogenous peroxidase was blocked with 3% H_2_O_2_ (DAKO) and slides were incubated for 30 min with primary antibodies to cleaved Caspase-3 (1/100, Asp 175, Cell Signaling Technology), or Myeloperoxidase (Neo Markers). A biotin-labelled secondary antibody was applied for 30 min, followed by avidin-biotin-peroxidase conjugate for 30 min. For visualisation, peroxidase enzyme substrate, 3,3′-diaminobenzidine was added and slides were counterstained with Harris haematoxylin. Positive cells were evaluated in 100 intestinal villi and 100 crypts located on each side of PP at high power magnification (×40).

### Flow cytometry analyses

PPs were removed, washed in PBS and cells were mechanically extracted and passed through a mesh filter. Cells were then centrifuged, washed in PBS and used for analysis. Staining was performed using the following antibodies (PE-Cy5-anti-CD3 (17A2), PE-Cy7-anti-CD3 (145-2C11), PE-anti-CD45R/B220 (RA3-6B2), according to manufacturer's protocols (BD Pharmingen). Ethidium homodimer-1 staining was performed using a commercially available kit (Molecular Probes). Data was acquired on a dual laser FACScan flow cytometer (Becton Dickinson) and analyzed using the Cell Quest 3.3 software (Becton Dickinson). Cell populations were gated on the basis of forward and side scatter to allow selection of the viable lymphocytes. Cells stained with 0.04% Trypan blue dye were counted in a hemocytometer.

### Ussing chamber experiments

Immediately after mouse sacrifice, a portion of jejunum containing a PP was opened along the mesenteric border and mounted in a 0.196 cm^2^ Ussing chamber. Throughout the experiment, tissues were maintained in circulating oxygenated Ringer solution at 37°C and the electric resistance was monitored. Bacteria (1×10^8^ CFU/ml) were added in the mucosal compartment of the chamber. After 120 min, a sample was taken out from the serosal side and the PP was removed, washed and homogenized. Bacteria from the serosal side and the PP tissue were counted on plated serial dilutions.

### In vitro cell culture experiments

Bone marrow-derived dendritic cells (BMDC) were obtained as previously described [33]. In brief, bone marrow was flushed from the femurs and tibias of mice. White blood cells were plated in IMDM medium (Biowhittaker) supplemented with 10% fetal calf serum (FCS) (Gibco), 1% supernatant from the J558 cell line [34], antibiotics (100 U of penicillin ml^−1^ and 100 µg of streptomycin ml^−1^) and 5×10^−5^ M 2-mercaptoethanol. On days 6 and 10, loosely adherent cells were collected and replated in fresh medium. Bone marrow-derived macrophages BMMP were prepared as previously described [35]. Briefly, cells obtained from the bone marrow were cultured for 5 to 6 days in DMEM medium (Seromed, Berlin, Germany) supplemented with 10% FCS (Dominique Dutscher, Brumath, France), 10% L-cell-conditioned medium, 100 U of penicillin ml^−1^, and 100 µg of streptomycin ml^−1^ at a concentration of 5×10^5^ cells ml^−1^.

Eighteen hours prior to infection, cells were washed with DMEM medium without antibiotics and were incubated in medium with 10% FCS and 5% L-cell-conditioned medium. Bacteria from fresh selective agar plates were inoculated into 2 ml LB medium with selection antibiotics and incubated over-night at 26°C with shaking. The number of bacteria was deduced of the optical density measure at 600 nm. 10^8^ bacteria were then added into 2 ml of complete cell culture medium without antibiotics and incubated 30 min at 26°C. After a temperature shift to 37°C, bacteria were then added to cell cultures at a calculated MOI of 10 in 12 well plates. To facilitate bacterial adhesion to the cells, the plates were centrifuged at 1300 rpm for 5 min. After 120 min, gentamycin (30 µg/ml) was added in order to kill extra-cellular bacteria. At 3 hours and 6 hours postinfection, the infected macrophages were washed gently three times with PBS and lysed by incubation in 0.5 ml of 0.1% Triton X-100 in PBS. After incubation for 15 min at 37°C, the lysates were removed and the wells were rinsed with 0.5 ml of PBS, and the lysates and rinses from each well were pooled. The number of released viable bacteria was determined by plating serial 10-fold dilutions on selective LB agar. For cytokine analyses supernatants were harvested 6 h after infection and cytokines were analyzes using commercially available Elisa Assays (BD Bioscience) according to manufacturer's instructions. For counts of cell death, cells were stained with 0.04% Trypan blue and counted in a hemocytometer.

### Statistical analysis

Two group comparisons were performed using unpaired t-test for data with Gaussian distribution and Mann-Whitney test if distribution was not Gaussian. Survival times were analyzed using Kaplan-Meyer curves and comparisons were performed using the Log-Rank test. Statistical analysis was performed using GraphPad Prism 4.00 (GraphPad Software, San Diego California USA) and SAS 8.02 (SAS, Cary, N.C., USA) software packages for PC. A value of P<0.05 was considered as statistically significant. All P values are two sided.

## Supporting Information

Figure S1 Survival curves of Nod2+/+ and Nod2−/− mice in the C57BL/6j and FVB/N backgrounds following orogastrically inoculation with 10 fold dilutions ranging from 6,5×105 to 6,5×109 CFU of Y. pseudotuberculosis YPIII(pIB102) strain (n = 5 Nod2+/+ and n = 5 Nod2−/− bmice for each dose group). Log-Rank test.
(0.12 MB TIF)

Figure S2 Bacterial counts in PP and organs of Nod2−/− mice (a–c) Nod2−/− (n = 15) and Nod2+/+ (n = 15) mice in the C57BL/6j background were orogastrically inoculated with 1×107 CFU of YPIII(pIB102) and bacterial counts in PPs, livers and spleens were analyzed at day 2. Nod2−/− had lower bacterial counts (P<0.05) in PPs than Nod2+/+ mice. No differences in bacterial counts were found in liver or spleen. (Mann Whitney test). Detection limit (DL) was 10^2^ CFU.
(0.09 MB TIF)

Figure S3 In vitro analyses of bone marrow derived macrophages and dendritic cells after Yersinia pseudotuberculosis infection. (a–f) Macrophages and dendritic cells were derived from bone marrow of Nod2−/− and Nod2+/+ mice. Cells were left uninftected (0) or were infected with Y. pseudotuberculosis YPIII(pIB102) (YP) at an MOI of 10. At 6 h post infection, secretion of TNFα and IL-1β by macrophages (a, b) and dendritic cells (c, d) of Nod2−/− cells did not differ from Nod2+/+ cells. (e) No differences were found for tryptan blue positive macrophages. (f) Gentamycine protection assays did not show differences of intracellular surviving bacteria in macrophages 3 h and 6 h after infection. (Student t-test). Data represent mean±SEM from three independent experimens (triplicate).
(0.11 MB TIF)

Figure S4 Nod2−/− mice are more susceptible to oral Salmonella typhimurium infection. Nod2−/− (n = 6) and Nod2+/+ (n = 6) mice in the C57BL/6j background were orogastrically inoculated with 5×108 CFU of S. typhimurium. Survival was found to be altered in Nod2−/− mice (P<0.05; Log-Rank test).
(0.05 MB TIF)

## References

[1] Inohara N, Nunez G (2003). NODs: intracellular proteins involved in inflammation and apoptosis.. Nat Rev Immunol.

[2] Hisamatsu T, Suzuki M, Reinecker HC, Nadeau WJ, McCormick BA (2003). CARD15/NOD2 functions as an antibacterial factor in human intestinal epithelial cells.. Gastroenterology.

[3] Rosenstiel P, Fantini M, Brautigam K, Kuhbacher T, Waetzig GH (2003). TNF-alpha and IFN-gamma regulate the expression of the NOD2 (CARD15) gene in human intestinal epithelial cells.. Gastroenterology.

[4] Ogura Y, Inohara N, Benito A, Chen FF, Yamaoka S (2001). Nod2, a Nod1/Apaf-1 family member that is restricted to monocytes and activates NF-kappaB.. J Biol Chem.

[5] Inohara N, Ogura Y, Fontalba A, Gutierrez O, Pons F (2003). Host recognition of bacterial muramyl dipeptide mediated through NOD2. Implications for Crohn's disease.. J Biol Chem.

[6] Girardin SE, Boneca IG, Viala J, Chamaillard M, Labigne A (2003). Nod2 is a general sensor of peptidoglycan through muramyl dipeptide (MDP) detection.. J Biol Chem.

[7] Abbott DW, Wilkins A, Asara JM, Cantley LC (2004). The Crohn's disease protein, NOD2, requires RIP2 in order to induce ubiquitinylation of a novel site on NEMO.. Curr Biol.

[8] Opitz B, Puschel A, Schmeck B, Hocke AC, Rosseau S (2004). Nucleotide-binding oligomerization domain proteins are innate immune receptors for internalized Streptococcus pneumoniae.. J Biol Chem.

[9] Kobayashi KS, Chamaillard M, Ogura Y, Henegariu O, Inohara N (2005). Nod2-dependent regulation of innate and adaptive immunity in the intestinal tract.. Science.

[10] Abbott M, Galloway A, Cunningham JL (1986). Haemochromatosis presenting with a double Yersinia infection.. J Infect.

[11] Heesemann J, Gaede K, Autenrieth IB (1993). Experimental Yersinia enterocolitica infection in rodents: a model for human yersiniosis.. Apmis.

[12] Brubaker RR (1991). Factors promoting acute and chronic diseases caused by yersiniae.. Clin Microbiol Rev.

[13] Autenrieth IB, Firsching R (1996). Penetration of M cells and destruction of Peyer's patches by Yersinia enterocolitica: an ultrastructural and histological study.. J Med Microbiol.

[14] Clark MA, Hirst BH, Jepson MA (1998). M-cell surface beta1 integrin expression and invasin-mediated targeting of Yersinia pseudotuberculosis to mouse Peyer's patch M cells.. Infect Immun.

[15] Handley SA, Dube PH, Revell PA, Miller VL (2004). Characterization of oral Yersinia enterocolitica infection in three different strains of inbred mice.. Infect Immun.

[16] Barreau F, Meinzer U, Chareyre F, Berrebi D, Niwa-Kawakita M (2007). CARD15/NOD2 is required for Peyer's patches homeostasis in mice.. PLoS ONE.

[17] Bolin I, Wolf-Watz H (1984). Molecular cloning of the temperature-inducible outer membrane protein 1 of Yersinia pseudotuberculosis.. Infect Immun.

[18] Maeda S, Hsu LC, Liu H, Bankston LA, Iimura M (2005). Nod2 mutation in Crohn's disease potentiates NF-kappaB activity and IL-1beta processing.. Science.

[19] Monack DM, Mecsas J, Bouley D, Falkow S (1998). Yersinia-induced apoptosis in vivo aids in the establishment of a systemic infection of mice.. J Exp Med.

[20] Cornelis GR, Boland A, Boyd AP, Geuijen C, Iriarte M (1998). The virulence plasmid of Yersinia, an antihost genome.. Microbiol Mol Biol Rev.

[21] Stainier I, Cornelis GR (1998). The Yop virulon of Yersinia: a bacterial weapon to kill host cells.. Clin Microbiol Infect.

[22] Heesemann J, Sing A, Trulzsch K (2006). Yersinia's stratagem: targeting innate and adaptive immune defense.. Curr Opin Microbiol.

[23] Fujimura Y, Kamoi R, Iida M (1996). Pathogenesis of aphthoid ulcers in Crohn's disease: correlative findings by magnifying colonoscopy, electron microscopy, and immunohistochemistry.. Gut.

[24] Meinzer U, Idestrom M, Alberti C, Peuchmaur M, Belarbi N (2005). Ileal involvement is age dependent in pediatric Crohn's disease.. Inflamm Bowel Dis.

[25] Van Kruiningen HJ, Ganley LM, Freda BJ (1997). The role of Peyer's patches in the age-related incidence of Crohn's disease.. J Clin Gastroenterol.

[26] Polito JM, 2nd, Childs B, Mellits ED, Tokayer AZ, Harris ML (1996). Crohn's disease: influence of age at diagnosis on site and clinical type of disease.. Gastroenterology.

[27] Saebo A, Vik E, Lange OJ, Matuszkiewicz L (2005). Inflammatory bowel disease associated with Yersinia enterocolitica O∶3 infection.. Eur J Intern Med.

[28] Ibbotson JP, Lowes JR, Chahal H, Gaston JS, Life P (1992). Mucosal cell-mediated immunity to mycobacterial, enterobacterial and other microbial antigens in inflammatory bowel disease.. Clin Exp Immunol.

[29] Lamps LW, Madhusudhan KT, Havens JM, Greenson JK, Bronner MP (2003). Pathogenic Yersinia DNA is detected in bowel and mesenteric lymph nodes from patients with Crohn's disease.. Am J Surg Pathol.

[30] Kallinowski F, Wassmer A, Hofmann MA, Harmsen D, Heesemann J (1998). Prevalence of enteropathogenic bacteria in surgically treated chronic inflammatory bowel disease.. Hepatogastroenterology.

[31] Swidsinski A, Ladhoff A, Pernthaler A, Swidsinski S, Loening-Baucke V (2002). Mucosal flora in inflammatory bowel disease.. Gastroenterology.

[32] Reed L (1938). A simple method for estimating fifty percent endpoints.. Am J Hyg.

[33] Mederle I, Bourguin I, Ensergueix D, Badell E, Moniz-Peireira J (2002). Plasmidic versus insertional cloning of heterologous genes in Mycobacterium bovis BCG: impact on in vivo antigen persistence and immune responses.. Infect Immun.

[34] Zal T, Volkmann A, Stockinger B (1994). Mechanisms of tolerance induction in major histocompatibility complex class II-restricted T cells specific for a blood-borne self-antigen.. J Exp Med.

[35] Jackson M, Phalen SW, Lagranderie M, Ensergueix D, Chavarot P (1999). Persistence and protective efficacy of a Mycobacterium tuberculosis auxotroph vaccine.. Infect Immun.

