# Supplementary figures and images for: Nod2 Mediates Susceptibility to Yersinia pseudotuberculosis in Mice
Source: PLoS One. 2008 Jul 23;3(7):e2769. doi: 10.1371/journal.pone.0002769 (PMC2447872; doi:10.1371/journal.pone.0002769)

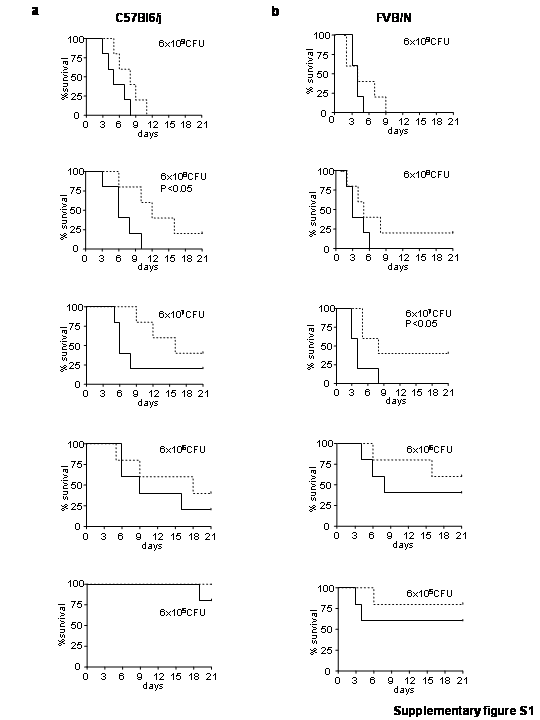

Supplement: Figure S1 — Survival curves of Nod2+/+ and Nod2−/− mice in the C57BL/6j and FVB/N backgrounds following orogastrically inoculation with 10 fold dilutions ranging from 6,5×10^5^ to 6,5×10^9^ CFU of Y. pseudotuberculosis YPIII(pIB102) strain (n = 5 Nod2+/+ and n = 5 Nod2−/− bmice for each dose group). Log-Rank test. (0.12 MB TIF) [file pone.0002769.s001.tif]

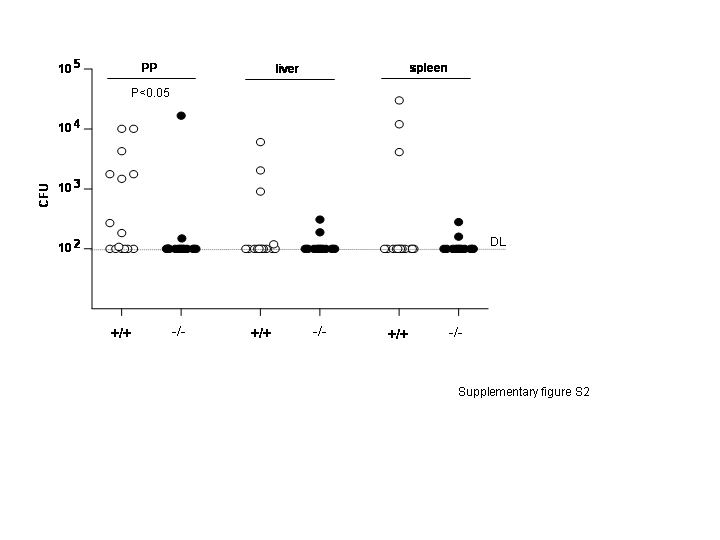

Supplement: Figure S2 — Bacterial counts in PP and organs of Nod2−/− mice (a–c) Nod2−/− (n = 15) and Nod2+/+ (n = 15) mice in the C57BL/6j background were orogastrically inoculated with 1×10^7^ CFU of YPIII(pIB102) and bacterial counts in PPs, livers and spleens were analyzed at day 2. Nod2−/− had lower bacterial counts (P<0.05) in PPs than Nod2+/+ mice. No differences in bacterial counts were found in liver or spleen. (Mann Whitney test). Detection limit (DL) was 102 CFU. (0.09 MB TIF) [file pone.0002769.s002.tif]

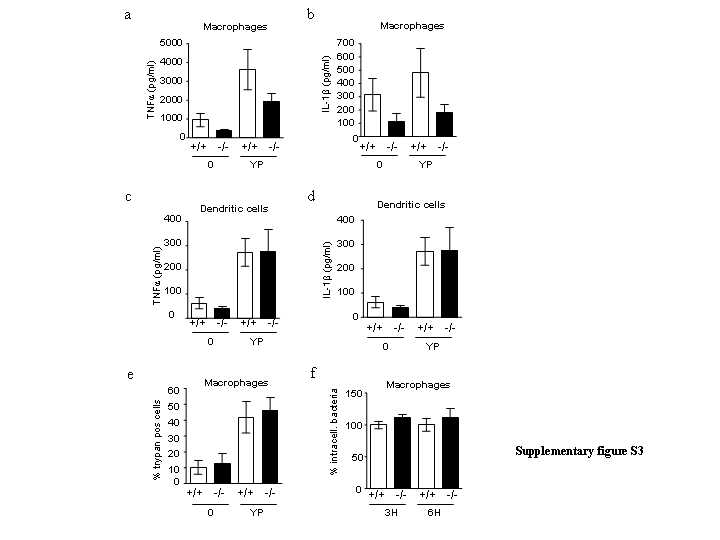

Supplement: Figure S3 — In vitro analyses of bone marrow derived macrophages and dendritic cells after Yersinia pseudotuberculosis infection. (a–f) Macrophages and dendritic cells were derived from bone marrow of Nod2−/− and Nod2+/+ mice. Cells were left uninftected (0) or were infected with Y. pseudotuberculosis YPIII(pIB102) (YP) at an MOI of 10. At 6 h post infection, secretion of TNFα and IL-1β by macrophages (a, b) and dendritic cells (c, d) of Nod2−/− cells did not differ from Nod2+/+ cells. (e) No differences were found for tryptan blue positive macrophages. (f) Gentamycine protection assays did not show differences of intracellular surviving bacteria in macrophages 3 h and 6 h after infection. (Student t-test). Data represent mean±SEM from three independent experimens (triplicate). (0.11 MB TIF) [file pone.0002769.s003.tif]

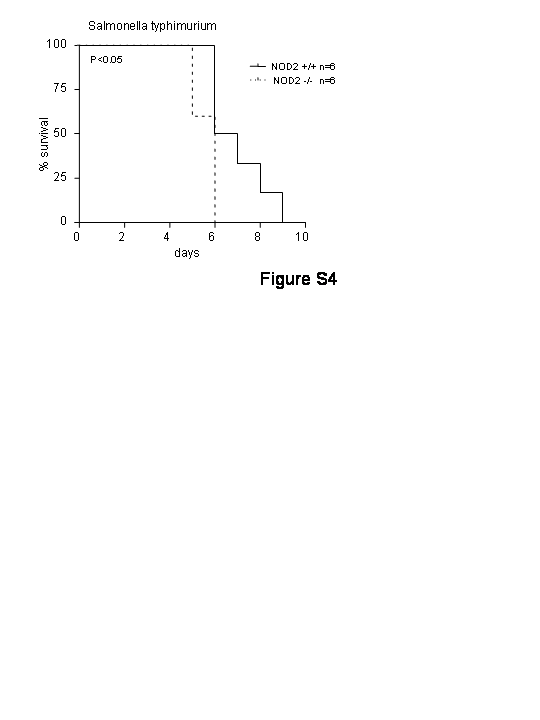

Supplement: Figure S4 — Nod2−/− mice are more susceptible to oral Salmonella typhimurium infection. Nod2−/− (n = 6) and Nod2+/+ (n = 6) mice in the C57BL/6j background were orogastrically inoculated with 5×10^8^ CFU of S. typhimurium. Survival was found to be altered in Nod2−/− mice (P<0.05; Log-Rank test). (0.05 MB TIF) [file pone.0002769.s004.tif]
